# BBSome: An essential component of hypothalamic regulation of energy homeostasis

**DOI:** 10.1007/s11154-025-09979-0

**Published:** 2025-06-25

**Authors:** Deng Fu Guo, Yuying Zhao, Connor Laule, Kamal Rahmouni

**Affiliations:** 1 Department of Neuroscience and Pharmacology, University of Iowa Carver College of Medicine, Iowa City, Iowa USA; 2 Veterans Affairs Health Care System, Iowa City, Iowa USA; 3 Fraternal Order of Eagles Diabetes Research Center, University of Iowa Carver College of Medicine, Iowa City, Iowa USA; 4 Obesity Research and Education Initiative, University of Iowa Carver College of Medicine, Iowa City, Iowa USA; 5 Department of Internal Medicine, University of Iowa Carver College of Medicine, Iowa City, Iowa 52242 USA

**Keywords:** Bardet-Biedl syndrome, Clia, Hypothalamus, Obesity, Metabolism

## Abstract

The BBSome is a protein complex composed of eight Bardet-Biedl syndrome (BBS) proteins. Although the BBSome is best known for its role in regulating cilia function, accumulating evidence indicates that this protein complex is also involved in various cellular processes, including mitochondrial dynamics and receptor trafficking to the plasma membrane. BBSome deficiency is associated with a range of diseases, including obesity and type 2 diabetes. Research into the mechanisms underlying metabolic disorders caused by BBSome deficiency has uncovered novel pathways of metabolic regulation, particularly within the hypothalamus. In this review, we discuss recent advances in understanding the role of the hypothalamic BBSome in the control of energy and glucose homeostasis and explore the underlying mechanisms.

## Introduction

Genetic studies have consistently pointed to the central nervous system (CNS) as a key player in the regulation of body weight and the development of obesity and adverse side effects [1, 2]. This is likely due to the tremendous influence the brain exerts on processes that control metabolism [3, 4]. The hypothalamus, which is a primary brain center for metabolic homeostasis, integrates and process signals related to energy stores leading to autonomic, humoral, and behavioral adaptations aimed at maintaining body weight homeostasis [5, 6]. Disruption in these systems can lead to metabolic disorders, particularly obesity, which is a significant public health issue worldwide.

Environmental factors—such as diet, physical activity, and lifestyle choices—play a crucial role in determining an individual's risk of developing obesity. However, for most individuals, obesity results from a complex interplay between these environmental influences and multiple genetic components. Genetic factors contribute significantly to body weight control by influencing how the body regulates hunger, energy storage, and metabolism. In fact, an estimated 40–70% of obesity risk is attributed to genetic heritability [7, 8]. For example, variations in the *FTO* gene have been associated with a higher risk of obesity, affecting hunger regulation and food preferences [9, 10].

Rare monogenic forms of obesity caused by mutations in a single gene, typically involved in the leptin-melanocortin system, have been instrumental in our understanding the fundamental mechanisms underlying the regulation of metabolic processes [12, 13]. Syndromic forms of obesity have also been valuable in gaining insights into the molecular mechanisms governing food intake and the neural networks that regulate ingestive behavior, satiety, and body weight [14]. Bardet-Biedl syndrome (BBS) is a rare autosomal recessive genetic disorder which typically results from a single gene mutation and affects 1:13,500 to 1:100,000 people around the world [15, 16]. BBS patients develop severe, early-onset obesity as a hallmark symptom [18]. This condition is common throughout the pediatric lifespan and affects more than 90% of individuals with BBS who are over 6 years old [19]. BBS is characterized by additional clinical features like type 2 diabetes, hypertension, retinal dystrophy or pigmentary retinopathy, intellectual disability, and renal abnormalities.

BBS exhibits substantial genetic heterogeneity, with over 26 genes implicated in the disorder [20]. Among these, *BBS1* is one of the most frequently mutated, with a single missense mutation—M390R—accounting for approximately 80% of all *BBS1*-related cases [21, 22]. This variant is particularly common in individuals of European and North American ancestry. Loss-of-function mutations are also frequently observed in *BBS2* and *BBS10* genes. Together with BBS1, these genotypes account for over 50% of affected individuals. Analysis of siblings and families of BBS patients revealed that heterozygosity of *BBS* gene mutations predispose individuals to excess weight gain and thus may contribute to common human obesity [23, 24]. Moreover, variants of several *BBS* genes including *BBS1*, *BBS2*, *BBS4*, and *BBS6* were found to increase susceptibility to obesity in non-BBS individuals [25, 26]. Additionally, *BBS* genes are linked to metabolic syndrome components like dyslipidemia and hyperglycemia, which are common complications in individuals with obesity [29]. The implication of *BBS* genes in polygenic human obesity suggests their broader role in metabolic regulation.

BBS proteins were originally associated with the function of cilia, which are small, hair-like structures found on the surface of many cells including neurons. This was due to the ciliary localization of BBS proteins and the disruption in cilia function in their absence. Eight BBS proteins (BBS1, BBS2, BBS4, BBS5, BBS7, BBS8, BBS9, and BBS18) interact to form a protein complex, termed the *BBSome.* This protein complex is essential for intraflagellar transport (IFT), which is critical for maintaining ciliary structure and signaling. However, extensive evidence points to a broader role for the BBSome through its implication as a critical regulator of various cellular processes such as plasma membrane localization of receptors, protein quality control, and metabolism, highlighting its importance beyond cilia-related functions [1]. In this review, we discuss recent progress in our understanding of the mechanisms by which the BBSome affects energy balance and its broader implications for understanding obesity and related metabolic disorders.

## Cellular functions of the BBSome

### BBSome assembly and structure

The discovery of the BBSome as a protein complex marked a pivotal moment in understanding the molecular basis of BBS because it explained how mutations in so many genes can lead to similar clinical phenotypes. The BBSome, which was identified by biochemical purification of BBS4-containing complexes from mammalian cells, was found to localize to non-membranous centriolar satellites in the cytoplasm and to the membrane of the cilium [30, 31]. These studies also revealed that the BBSome has a unique multi-protein assembly, resembling a"coat complex"that surrounds and regulates the trafficking of ciliary membrane proteins. The BBSome was found to assemble in a regulated, stepwise process. Initially, BBS2 and BBS7 combine to form a foundational structure, followed by the addition of BBS9, contributing to the formation of the core complex. Subsequently, the core is augmented with the incorporation of BBS5 and BBS8, leading to a complete BBSome assembly. It is noteworthy that functional subcomplexes can form when genes encoding BBSome components are mutated [32, 33]. For instance, in the absence of BBS8, a subcomplex comprising BBS2, BBS5, BBS7, and BBS9 can still form [34]. This ability to form subcomplexes suggests a level of structural redundancy within the BBSome, allowing it to partially compensate for certain genetic mutations.

**Fig. 1:**
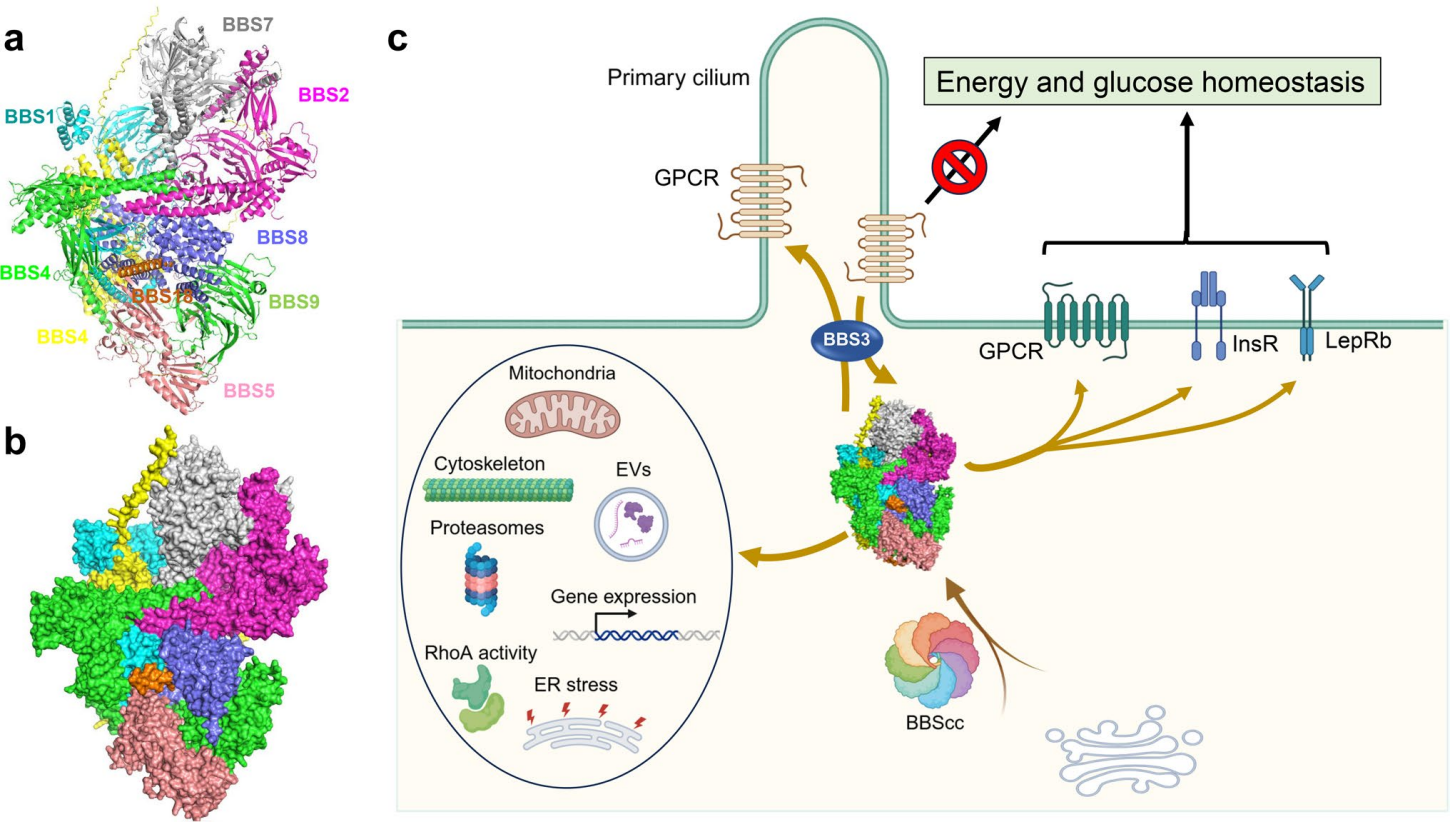
Overall structure of the BBSome and its cellular functions. **a**, **b** Structural views of the human BBSome: **a** predicted model and **b** surface representation (both generated using the AlphaFold3 Model 1). **c** Schematic representation of the various roles of the BBSome in the control of different cellular functions including trafficking of cargos to cilia and plasma membrane (created using BioRender). The assembly of BBSome subunits is facilitated by the BBS chaperonin complex (BBScc), which includes BBS6, BBS10, and BBS12 proteins. BBSome impact on metabolic functions relate to its role in trafficking various receptors (e.g. GPCRs, insulin receptor (InsR) and leptin receptor (LepRb)) to the plasma membrane, but not to cilia

BBSome structure has been solved using high-resolution electron cryomicroscopy that revealed how the subunits interact with each other (Fig. 1a–b). These studies demonstrated that the BBSome consists of three main regions: the head, where BBS2 and BBS7 are located; the body lobes, composed of BBS1, BBS4, BBS8, BBS9, and BBS18; and the tail, formed by BBS5. BBS1 structurally links the head and body lobes, with its β-propeller domain acting as a central scaffold. Additionally, BBS9 and a helical neck—formed by adjacent coiled coils from BBS2—further stabilize the connection between the head and body.

Beyond the BBSome core subunits, other BBS proteins are important for BBSome formation. In particular, BBS6, BBS10, and BBS12 interact with CCT/TRiC chaperonin proteins to form another complex, termed BBS chaperonin complex (BBScc), which facilitates initial BBSome assembly in an ATP-dependent manner [35] (Fig. 1c). Moreover, BBSome assembly is regulated by the BBS3 (aka small Arf-like GTPase ARL6), to produce the BBSome coat complex (outside of the core subcomplex) critical for membrane targeting [36]. High-resolution analysis showed that the BBSome adopts an open conformation, allowing it to bind the membrane-associated GTPase BBS3 [37, 38]. A large, positively charged patch on the BBSome likely enhances its interaction with the membrane. Interestingly, BBSome activation appears to require an unexpected swiveling of the BBS1 β-propeller domain—the subunit most frequently involved in substrate recognition—which expands the central cavity of the complex. Crystal structures showed that GTP–BBS3 binds to the BBS1 β-propeller at blades 1 and 7, explaining why the GTP-bound form, but not GDP-bound, BBS3 can recruit the BBSome to membranes [36]. Single-point mutations at the BBS3–GTP–BBS1 interface disrupt this interaction, preventing BBSome import into cilia. Additionally, the M390R mutation in the *BBS1* gene impairs its ability to interact with BBS–GTP, demonstrating how disease-causing mutations hamper this interaction, leading to pathologies.

### BBSome function in cilia

The BBSome is well-known for mediating ciliary transport by facilitating certain protein cargo trafficking primarily through interactions with the IFT machinery, which consists of IFT-A and IFT-B complexes [39]. The ciliary membrane contains various receptors that modulate intraciliary signaling molecules such as Ca^2+^, cAMP, and membrane potential changes via ion channels, enabling transmission of extracellular signals. These signals are transduced into cells, affecting a wide range of cellular processes, including gene transcription, cell division, and differentiation [40].

The BBSome, as an adaptor complex, manages the entry and exit of signaling receptors and other proteins into the cilia, with IFT proteins (Fig. 1c). As noted above, membrane recruitment of the BBSome requires the ARL6/BBS3 for entry to cilia [41]. Upon GTP binding, BBS3 associates with the membrane and recruits the BBSome to target membrane proteins to cilia. Interestingly, BBS3 has also been reported to promote BBSome-mediated exit of specific cargo from cilia [42]. BBS17 determines BBSome availability for integration into anterograde IFT trains to enter cilia by facilitating BBS3 targeting to the basal body of a cilium [43, 44]. Surprisingly, although BBSome disruption leads to subtle ciliary changes, the BBSome is not required for cilia formation [41, 45, 46]. The BBSome is involved in trafficking of several proteins to cilia, such as polycystin-1 [33] and various G protein-coupled receptors (GPCRs) including dopamine receptor 1, somatostatin receptor 3 (SSTR3), melanin concentrating hormone receptor 1 (MCHR1), and neuropeptide Y2 receptor (NYP2R) [48, 49] (Fig. 1c).

While the BBSome is primarily known for its role in anterograde transport, substantial evidence underscores its importance for retrograde trafficking, working in coordination with the IFT-A system and the dynein motor complex to retrieve and recycle ciliary proteins following activation. This process ensures the proper removal of proteins and prevents ciliary accumulation of signaling receptors in the cilium [51]. This finding is supported by accumulation in cilia of certain GPCRs such as Smoothened [52], patched 1 [52], dopamine receptors [32], and GPR161 in BBS1-deficient cells. BBSome-mediated GPCRs’ retrograde transport is initiated by GPCR kinase 2, by phosphorylating activated GPCRs [53, 54]. β-arrestin then binds to the phosphorylated GPCRs and adds ubiquitin to the receptor C-terminus. Ubiquitinylated GPCRs are recognized by TOM1-like protein 2 to facilitate removal from cilia [55, 56]. BBS3 is necessary for retrieval of activated SSTR3, and BBS3 has been shown to mediate BBSome ciliary turnover by promoting its outward movement across the transition zone in the Chlamydomonas system [58].

In addition to retrieval via the BBSome-mediated trafficking pathway, cells can also limit the accumulation of signaling proteins in the primary cilium through the shedding of ectosomes from the ciliary tip [59, 60]. This process involves the outward budding and release of small vesicles—ectosomes—that carry excess or mislocalized ciliary membrane proteins, including signaling receptors. In the context of BBSome dysfunction, when retrograde transport is impaired and retrieval of proteins from the cilium is compromised, ectosomal shedding serves as an alternative clearance mechanism to preserve ciliary composition and prevent aberrant signaling [61]. This compensatory process highlights the cilium’s dynamic regulation and underscores the importance of maintaining its proteomic integrity.

### BBSome and receptor trafficking to plasma membrane

Research into the mechanisms driving obesity associated with BBS has revealed a critical function for the BBSome in the proper localization of various receptors on the cell surface. A key BBSome component (BBS1) was found to physically associate with the leptin receptor (LepRb), facilitating its transport to the plasma membrane [62] (Fig. 1c). No other BBSome subunits were found to interact with the LepRb. The specific C-terminal cytoplasmic segment of LepRb was sufficient for binding to BBS1. Furthermore, the *BBS1 M390R* mutation significantly impaired the ability of BBS1 to bind to LepRb. On the other hand, BBS10 was found to enhance the stability of LepRb, possibly by boosting its synthesis and/or decreasing its degradation [63]. Remarkably, disrupting the BBSome substantially diminished surface expression of LepRb. This was associated with leptin resistance caused by blunted leptin activation of signal transducer and activator of transcription 3 (Stat3), a downstream effector of the LepRb [64, 65]. BBSome trafficking of the LepRb operates independently of cilia, as removing cilia (via Ift88 gene deletion) did not reproduce the leptin resistance observed by BBSome disruption. In addition to the LepRb, the BBSome has been implicated in the trafficking and plasma membrane localization of several other receptors including the insulin receptor [63, 66], Notch receptor [67], and serotonin 5-hydroxytryptamine 2 C receptor (5-HT2CR) [68]. Taken together, these findings suggest that disrupted trafficking and signaling of critical receptors as the molecular mechanism of metabolic disorders associated with BBS.

The fact that BBSome deficiency reduces membrane localization of various receptors without altering their total expression raised the possibility that BBS proteins may interact with components of the secretory pathway to ensure proper vesicular transport between organelles, such as the endoplasmic reticulum and Golgi apparatus. Consistent with such a possibility, BBSome components were found to colocalize with late endosomes, but not with the early endosomes or the Golgi apparatus [68]. This observation suggests that the BBSome helps modulate the transport of materials between different compartments within the cell, which is essential for processes like membrane trafficking, cell signaling, and maintaining cellular homeostasis. Indeed, BBSome disruption causes accumulation of 5-HT2CR in late endosomes. It should be noted that in zebrafish BBSome depletion inhibits the retrograde transport of melanosomes, further confirming the importance of BBS proteins for intracellular organization and function [69]. These findings underscore the essential role of the BBSome in maintaining organelle integrity and regulating intracellular trafficking.

### BBSome regulation of mitochondria function

A study by Gohlke et al. demonstrated that loss of the *Bbs4* gene in mice results in metabolic inefficiency and impaired lipid metabolism, both closely linked to mitochondrial dysfunction [70]. Indeed, *Bbs4* knockout mice exhibited cold intolerance and defective thermogenesis, supporting a role for the BBSome in mitochondrial function. In a mouse immortalized collecting duct cell line (IMCD3), deletion of the *Bbs10* gene led to intracellular lipid accumulation and metabolic abnormalities, accompanied by evidence of mitochondrial dysfunction [71]. However, it remains unclear whether mitochondrial dysfunction is a cause or a consequence of lipotoxicity in this context. Nonetheless, BBS10 was found to colocalize with mitochondrial markers, and six mitochondrial proteins were identified among its interactors [71].

More recent studies have directly implicated the BBSome in the regulation of mitochondria structure and function [72, 73]. Specifically, loss of BBSome function through deletion of *Bbs1*, *Bbs4,* or *Bbs8* gene was found to cause defects in mitochondrial morphology as indicated by increased mitochondrial length in various BBSome-deficient cells and tissues including mouse hypothalamic N39 cells, IMCD3 cells, CNS neurons, and brown adipose tissue. These morphological alterations translate into functional abnormalities, characterized by reduced oxygen consumption rate, altered mitochondrial distribution, and Ca^2+^ signaling [72]. Mechanistically, the BBSome was shown to modulate activity of dynamin-like protein 1 (DRP1), a key regulator of mitochondrial fission. Furthermore, the decrease in DRP1 activity, and the defects in the mitochondrial morphology and function evoked by BBSome deficiency can be mitigated through partial loss of a DRP1 inhibitor, A-kinase anchoring protein. Whether restoring mitochondrial dynamics reverses the defects in receptor trafficking was also examined. Interestingly, insulin receptor localization in the plasma membrane was significantly higher in BBS1-deficient cells in which mitochondrial function was restored with DRP1S637A, a constitutively active form of DRP1 [73]. These results implicate mitochondrial defects in plasma membrane receptor mislocalization associated with BBSome deficiency. However, significant gaps remain in understanding the relationship between the BBSome and mitochondria. For instance, the precise molecular link between the BBSome and DRP1 activity, and how this interface integrates with other known roles of the BBSome in membrane trafficking and signaling, warrants further investigation.

### Other cellular functions of the BBSome

The BBSome has been shown to regulate many other cellular processes (Fig. 1c). For instance, this protein complex influences intracellular processes involved in cell migration and tissue repair, as its deficiency causes an increase in RhoA expression and activity, along with a decrease in the ubiquitin ligase Culin-3, leading to impaired cell migration and wound healing [74]. Furthermore, the BBSome has been found to play a role in cytoskeleton organization, cell division, and the endoplasmic reticulum stress response [75, 76]. It remains unclear whether the BBSome's regulation of functions like actin cytoskeleton dynamics is connected to its role in cilia.

The BBSome has been reported to modulate release of bioactive extracellular vesicles (EVs) [79]. These EVs contains an abundance of Wnt-related molecules selectively secreted via distinct modes of EV biogenesis coordinated via the cilium. Given the emerging disease role of EVs and their potential use as novel therapeutic approaches to treat various diseases including metabolic disorders, further work is needed to better understand the role of the BBSome in the regulation of EVs release.

The BBSome also modulates proteasomal function because reduction in levels of BBS1 or BBS4 alters the composition of proteasomes, consequently inhibiting their activity [80]. Moreover, BBS7 interacts with nuclear proteins, suggesting a role for BBSome proteins in the nucleus. Particularly, BBS7 has been identified as a regulator of the nuclear protein RNF2, indicating a possible influence on transcriptional regulation [81]. It should be noted that the BBSome may also contribute to neuronal functions, such as synaptic plasticity and activity crucial for metabolic physiology [82]. Taken together, this evidence points to multiple functions of the BBSome and BBS proteins in the regulation of cellular processes through mechanisms that involve cilia or not. This complexity highlights the need for further research to fully elucidate the diverse role of the BBSome in cellular biology.

## Peripheral and neuronal substrates for BBSome regulation of metabolism

**Fig. 2:**
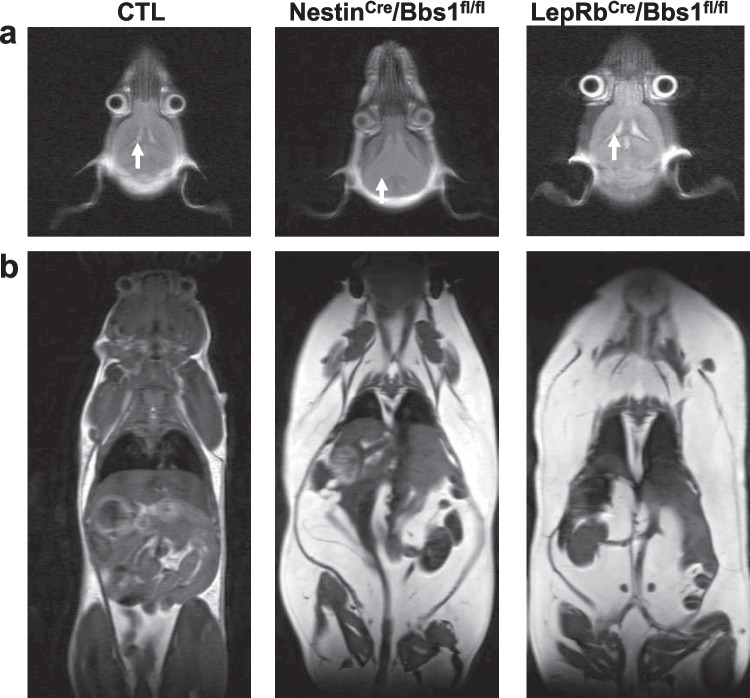
Obesity caused by neuronal BBSome deficiency is independent of hydrocephalus. Representative magnetic resonance images showing enlarged brain ventricles (arrow) in mice lacking the *Bbs1* gene in the nervous system, but not in LepRb neurons **a** whereas body fat (bright white area in images, **b** was elevated in both mouse models relative to control mice (CTL)

Mice bearing global deletion of several *Bbs* genes including *Bbs2*, *Bbs4*, *Bbs6*, *Bbs7*, *Bbs8*, *Bbs10*, or *Bbs1 M390R* mutation recapitulate many of the phenotypes observed in BBS patients, including obesity [32, 83]. Interestingly, relative to littermate wildtype controls, BBS mice are smaller at weaning, but their growth catches up around 8 weeks and they subsequently become significantly heavier than their control littermates around 12 weeks [45, 84]. The mechanism(s) underlying reduced size at birth remain unclear but may be due to altered cell cycle processes and cell proliferation [74]. Generally, obesity is more pronounced in female global BBS null mice than the male counterparts. It should be noted that in addition to metabolic disorders, BBS mice recapitulate many of the BBS phenotypes including the development of hydrocephalus (Fig. 2a). However, while human BBS patients typically develop polydactyly, this phenotype is not observed in global BBS knockout mice. The underlying mechanisms of these discrepancies remain to be elucidated.

### Peripheral BBSome and metabolic homeostasis

Earlier evidence implicated BBS proteins in adipocyte differentiation and function. For instance, *BBS* genes showed a temporal and synchronized expression during adipogenesis [85]. In 3T3 preadipocytes, which possess primary cilia, silencing the *Bbs4* gene accelerated cell division, leading to aberrant differentiation and excessive accumulation of triglycerides [86]. Conversely, overexpression of *Bbs4* in preadipocytes decreased proliferation rate. Furthermore, *Bbs12* gene deletion in pre-adipocytes was reported to alter adipogenesis by causing hyperplasia and hypertrophy [87]. These studies suggest a role for BBS proteins in early stages of adipocyte development, coinciding with the presence of primary cilia. Moreover, BBS proteins have been implicated in the regulation of adipose tissue lipid metabolism. Cold exposure in *Bbs4* null mice revealed impaired lipid utilization within adipose tissue, leading to an increased reliance on systemic lipid mobilization to meet the heightened energy demands associated with reduced body temperature [70].

To further investigate the significance of the BBSome in adipocytes, which in contrast to preadipocytes are not equipped with cilia, the effects of selective deletion of the *Bbs1* gene was examined (Table 1). Surprisingly, BBSome disruption in mature adipocytes had no effect on body weight although it altered glucose homeostasis and insulin sensitivity [88]. This phenotype appears to result from defective insulin receptor signaling in adipose tissue, consistent with a critical role for the BBSome in insulin action [89] and insulin receptor trafficking [66].

**Table 1:** Effect of BBSome deficiency through *Bbs1* gene deletion across different cell types and tissues on the development of obesity and type 2 diabetes phenotypes in mice

BBSome deficiency in	Obesity	Diabetes
Nervous system	Yes	Yes
LepRb neurons	Yes	Yes
POMC neurons	Yes	Yes
AgRP neurons	Yes	Yes
SF1 neurons	Yes	Yes
Dopamine neurons	Yes	Yes
Adipocytes	Yes	Yes
Skeletal muscle cells	Yes	Yes
Hepatocytes	Yes	Yes
Endothelial cells	Yes	Yes
Smooth muscle cells	Yes	Yes
Immune cells	Yes	Yes

The importance of the BBSome in other insulin-sensitive tissues was also examined. Absence of the BBSome in skeletal muscle failed to interfere with body weight regulation [73]. Notably, chow-fed female, but not male, mice lacking the BBSome in myocytes exhibited a tendency towards insulin intolerance, whereas when fed an obesogenic high-fat high sucrose diet males, but not females, displayed significantly increased insulin sensitivity during an insulin tolerance test [73]. This indicates that the BBSome in skeletal muscle promotes insulin resistance in diet-induced obesity. BBSome deficiency in hepatocytes increased body weight and adiposity, and it recapitulated hepatic steatosis observed in BBS patients [73, 90]. The moderate obesity observed in mice lacking the hepatic BBSome was not associated with hyperphagia, but these mice had impaired glucose handling and insulin resistance due to compromised insulin signaling in hepatocytes [73], underscoring the relevance of the hepatic BBSome for metabolic regulation.

In addition to insulin-sensitive tissues, the contribution to metabolic regulation of the BBSome in other tissues and cell types was also investigated. Unexpectedly, BBSome disruption in endothelial cells increased body weight and fat mass and caused hepatic steatosis associated with dysregulation in lipid profile [91]. While under normal chow-fed condition, male mice bearing BBSome deficiency in endothelial cells showed a minor impairment in insulin sensitivity when fed an obesogenic diet; these male mice displayed an improvement in glucose handling despite increased body weight. These findings indicate that the endothelial BBSome promotes insulin resistance in obesity. On the other hand, loss of the BBSome in smooth muscle cells or immune cells did not interfere with metabolic regulation [92, 93]. Collectively, these findings point to the relevance of the BBSome in certain peripheral cells and tissues, particularly hepatocytes and endothelial cells, for the regulation of body weight, glucose handling, and insulin resistance. It should be noted, however, that the frank obesity observed in global BBS mice was not recapitulated by BBSome disruption in any of the peripheral cells and tissues.

### Control of body weight by the hypothalamic BBSome

The hypothalamus is known to play a pivotal role in maintaining energy balance by balancing energy intake and expenditure [5]. Within the hypothalamus, several nuclei and neuronal populations have been implicated in the control of feeding behavior and energy expenditure. The arcuate nucleus of the hypothalamus contains two established antagonistic neuronal populations: anorexigenic proopiomelanocortin (POMC) neurons and orexigenic neurons expressing agouti-related peptide (AgRP) [94]. Both neuron types express several receptors essential for the regulation of metabolic homeostasis, including LepRb, insulin receptor, and GPCRs such as NPY2R and ghrelin receptors. Their strategic location adjacent to the median eminence enables these neurons to directly sense circulating factors without crossing the brain-blood barrier. Collectively, they play a critical role in controlling feeding behavior and energy expenditure, acting as first-order neurons that mediate the effects of various signals, such as leptin, insulin, ghrelin, and serotonin. Nearby neurons in the hypothalamic ventromedial nucleus that express steroidogenic factor 1 (SF1) have also been implicated in the control of various physiological functions including energy and glucose metabolism [95, 96].

To investigate the relevance of the neuronal BBSome to metabolic regulation this complex was disrupted in the nervous system by crossing Nestin^Cre^ mice with *Bbs1* floxed mice (Table 1). Importantly, *Bbs1* gene deletion from the nervous system interrupted neuronal BBSome function and largely recapitulated the obesity phenotype of BBS, where both male and female mice bearing nervous system *Bbs1* gene deletion exhibited significantly higher body weight and fat mass than their littermate controls [65]. The increased adiposity was more obvious in female mice lacking *Bbs1* gene in the nervous system than the male counterparts, indicating sex difference. The observed obesity in these mice is associated with hyperphagia, which may be due to reduced mRNA levels of the anorexigenic neuropeptide POMC despite normal AgRP and NPY mRNA levels.

The significance of the BBSome in specific neuronal populations for metabolic homeostasis was also examined. Mice harboring *Bbs1* gene deletion specifically in LepRb-expressing neurons, the majority of which residing in the hypothalamus, gained significantly more weight than littermate controls [65]. This observation is linked to hyperphagia and reduced energy expenditure as indicated by the increased food intake and decreased oxygen consumption and heat production. To dissect the contribution of hyperphagia versus energy expenditure to the development of obesity in the conditional BBS mouse models, pair-feeding experiments were performed. When mice lacking the *Bbs1* gene in LepRb-expressing cells were fed the same amount of food consumed by controls, it was found that hyperphagia plays a larger role in the development of obesity compared to energy expenditure [65]. Approximately 2/3 of the body weight gain in these mice was due to increased food intake, and about 1/3 was due to reduced energy expenditure.

Mice lacking the *Bbs1* gene in LepRb-expressing cells exhibited leptin resistance as indicated by elevated endogenous leptin levels and a blunted response to exogenous leptin, even when body weight was normalized by caloric restriction (beginning at 4–5 weeks until 12–14 weeks of age) [65]. It should be noted that lean global BBS null mice that had their body weight and adiposity normalized by calorie restriction also display leptin resistance [62]. Together, these findings—which are consistent with the requirements of BBS1 protein for the LepRb trafficking to the plasma membrane discussed above—indicate that obesity in BBS is mediated, at least in part, by impaired LepRb trafficking.

Fasting induced opposing changes in the expression of several *BBS* genes in POMC and AgRP neurons, highlighting their sensitivity to energy status [68]. Moreover, BBSome disruption in POMC or AgRP neurons caused obesity as indicated by the elevated body weight and adiposity. However, hyperphagia was only observed in mice with POMC neuron–specific *Bbs1* gene deletion. The expression of hypothalamic anorexigenic and orexigenic genes yielded noticeable differences. Only *Pomc* mRNA was decreased in mice with BBSome disruption in POMC neurons, whereas there were no transcript alterations in mice with AgRP neuron-specific BBSome disruption. This suggests a distinct molecular mechanism underlying obesity caused by BBSome dysfunction in POMC and AgRP neurons. Obesity in animals with AgRP neuron–specific *Bbs1* gene deletion could be due to altered energy expenditure and/or nutrient partitioning [98, 99].

Interestingly, deletion of the *Bbs1* gene in dopamine D1 receptor–containing neurons caused significant weight gain in mice starting at 8–10 weeks of age [100]. These mice showed normal feeding behavior but displayed significantly lower levels of basal locomotor activity that was not caused by defect in gross motor skill. These phenotypes may be due to the reduced D1-dependent signaling caused by accumulation of this receptor in cilia when the BBSome is absent [100].

Disruption of the BBSome in SF1 neurons leads to higher body weight and adiposity associated with reduced energy expenditure without altering food consumption [97]. Notably, mice lacking the *Bbs1* gene in SF1 neurons exhibited lower brown adipose tissue heat production, which was supported by a reduced level of thermogenic uncoupling protein 1, as well as significantly lower brown adipose tissue sympathetic nerve traffic. In addition, SF1^Cre^/Bbs1^fl/fl^ mice have reduced sympathetic traffic to white adipose tissue coupled with a protein expression profile that promotes lipid storage. This suggests that the ventromedial hypothalamus BBSome is important for regulation of metabolic homeostasis through the sympathetic nervous system. The ventromedial hypothalamus BBSome has also been implicated in dietary obesity–associated central resistance to bone morphogenic protein 8B (BMP8B), a member of the transforming growth factor beta-BMP superfamily [101]. This is supported by reduced BBS1 protein levels in the ventromedial hypothalamus of diet-induced obese rats and after BMP8B administration. The fact that resistance to central BMP8B is recapitulated in Bbs1 M390R mice demonstrates that ventromedial hypothalamus BBSome deficiency evoked by reduction in BBS1 levels/function is associated with resistance to BMP8B. However, the exact molecular mechanisms underlying this effect requires further investigation.

In BBS, hydrocephalus, although less common, can occur due to defects in motile cilia function that are critical for cerebrospinal fluid circulation, potentially leading to an accumulation of fluid in the brain [45]. Hydrocephalus can cause swelling and pressure in the brain, potentially affecting the hypothalamus and the mechanisms that regulate feeding behavior and energy expenditure [102]. However, hydrocephalus developed only in obese mice lacking the *Bbs1* gene in the nervous system, but not in other neuron-specific conditional null mice such as those lacking the BBSome in the LepRb or POMC neurons, which are also obese (Fig. 2). This indicates that obesity in BBS is not related to the development of hydrocephalus.

### Regulation of glucose homeostasis by the hypothalamic BBSome

Type 2 diabetes and associated abnormalities in glucose metabolism and insulin sensitivity are common in BBS patients [103, 104]. Insulin resistance, hyperinsulinemia, and impaired glucose tolerance are often present in early childhood [104, 106]. Feuillan et al. demonstrated that even when matched for pubertal stage and body composition, individuals with BBS are more insulin-resistant than controls [107], supporting the notion that insulin resistance associated with BBS is a primary defect. Importantly, BBS mice recapitulate these diabetes-related phenotypes as indicated by significantly elevated blood glucose and plasma insulin in these animals [66]. Moreover, glucose and insulin tolerance tests showed that BBS mice are glucose-intolerant and insulin-resistant. This was associated with reduced insulin receptor signaling. Notably, lean BBS mice that had their body weight and adiposity controlled by calorie restriction displayed significantly elevated plasma insulin and blunted insulin receptor signaling, indicating that insulin resistance associated with BBS is independent of obesity.

As mentioned above, POMC and AgRP neurons sense glucose from circulation and signal to metabolic tissues such as skeletal muscles, adipose tissues, and the liver to regulate systemic glucose levels. *In vivo* studies found that BBSome disruption in POMC neurons impaired glucose handling associated with a trend towards insulin resistance [108]. While glucose and insulin tolerance has not been directly tested in mice lacking the *Bbs1* gene in AgRP neurons, these animals have elevated insulin levels, suggesting a role in glucose homeostasis. Remarkably, despite obesity, mice bearing *Bbs1* gene deletion in SF1 neurons tended to be glucose-intolerant, whereas insulin sensitivity was unchanged [97]. These studies highlight the distinct role of the BBSome in different hypothalamic neuronal populations involved in glucose regulation and warrant further investigation into its effects on other neuronal groups.

## Mechanisms underlying hypothalamic BBSome regulation of metabolism

Regulation of metabolic and glucose homeostasis by the BBSome has been attributed to impaired cilia function. Chemical synapses and axonal segments have been found adjacent to neuronal cilia that express neuromodulatory receptors, supporting a direct role for cilia in neuronal communication [109]. Loss of neuronal cilia in mice leads to hyperphagia and obesity [110, 111]. The BBSome regulates protein transport to cilia, including receptors and other signaling proteins implicated in hypothalamic regulation of body weight and energy balance [112]. Loss of the functional BBSome affects trafficking of several GPCR ciliary proteins mediating energy homeostasis including SSTR3, MCHR1, and NYP2R [49, 50]. Together, these findings indicate that BBSome-mediated ciliary targeting of GPCR is important for energy balance. It should be noted, however, that very few ciliopathies are associated with metabolic perturbations. Indeed, among the various cilia-related diseases only patients with BBS and Alström syndrome develop obesity [29]. Consistent with this, loss of cilia in the LepRb neurons through *Ift88* gene deletion caused marginal or no change in body weight and adiposity [65, 113]. Moreover, loss of ciliary localization of the BBSome through deletion of BBS3 has an unimpressive impact on energy balance. For instance, global Bbs3 knockout mice have a minimal increase in body weight and adiposity compared to mice lacking BBSome components which develop frank obesity [114]. Food intake measurements showed that Bbs3 null mice are not hyperphagic, unlike other BBS knockout mice [45, 62, 64]. Thus, loss of BBS3 barely recapitulates metabolic alterations evoked by BBSome deficiency. Consistent with this, most BBS3 patients of Northern European descent exhibited little to no obesity phenotype [115]. It should be noted, however, that BBS3 patients in Arab Bedouin and Iranian families develop obesity [116, 117], underscoring the variability within and among families on the prevalence and severity of obesity in BBS3 patients.

A recent study provided strong evidence that regulation of energy homeostasis by the BBSome is not linked to its ciliary function. Interfering with BBSome localization in cilia by deleting BBS3 in POMC neurons had no effect on body weight and fat mass, despite reducing NPY2R trafficking to cilia [108]. This shows that the contribution of the BBSome to energy homeostasis is independent of its interaction with BBS3 and, by extension, from its ciliary function. Rather, the BBSome appears to influence body weight by regulating plasma localization of key receptors involved in energy homeostasis such as the LepRb, insulin receptor, and 5-HT2CR (Fig. 1c). Notably, BBS17 was found to play a role in leptin sensitivity by influencing the activation of the transcription factor Stat3 by the LepRb.

Wang et al. used induced pluripotent stem cell-derived hypothalamic arcuate-like neurons (iPSC-derived) to demonstrate that BBS1M390R and BBS110C91fsX95 mutated hypothalamic neurons have compromised leptin and insulin signaling in human fibroblast and iPSC-derived neurons [63]. This associated with decreased levels of POMC expression and neuropeptide production in mutated human fibroblasts and hypothalamic neurons. Furthermore, correcting these *BBS* gene mutations rescued leptin signaling. These findings show that defective leptin receptor and insulin receptor trafficking and subsequent signaling resistance, likely translates to humans.

As discussed above, the BBSome has emerged as an important regulator of mitochondria function. The relevance of this observation to the phenotypes associated with BBS including obesity was underscored by the demonstration that rescuing mitochondria defects evoked by BBSome deficiency significantly attenuated excess weight gain and adiposity [72]. These findings highlight defects in mitochondrial function as a critical mechanism underlying BBS pathology and raise the interesting possibility that rescuing mitochondria dysfunction may be beneficial for the management of BBS-associated pathologies. Additionally, BBS1-null cells can serve as a valuable tool for screening small molecules that enhance DRP1 activity and improve mitochondrial function, potentially paving the way for novel BBS treatments. However, the connection between the BBSome and DRP1 remains unclear. Additionally, further investigation is needed to understand how elongated mitochondria influence trafficking and localization of metabolic receptors.

## Conclusions and perspectives

The BBSome's role in cellular and physiological regulation extends beyond its ciliary function. The involvement of BBS proteins in metabolic control is linked to the essential effect of the BBSome on key cellular pathways that maintain organelle integrity and intracellular trafficking, particularly the transport and plasma membrane localization of receptors critical for energy homeostasis such as the LepRb, insulin receptor, and 5-HT2CR. The recognition of hypothalamic leptin signaling as a major defect causing energy imbalance in BBS [62, 64, 68] has led to the development of a drug that targets the downstream melanocortin system (setmelanotide) as an effective strategy to manage obesity in BBS patients [118].

While both peripheral and CNS BBSome function contribute to metabolic regulation and disorders, the hypothalamic BBSome more directly and potently influences metabolic regulation. These findings implicate hypothalamic neurons as the origin of obesity in BBS. Moreover, identification of the hypothalamic BBSome dysfunction in diet-induced obese animals [101] point to the involvement of BBS proteins in the development of common obesity, which is in line with the genetic findings implicating polymorphism in *BBS* genes in polygenic human obesity [29]. Thus, deciphering mechanisms underlying the contribution of *BBS* genes and proteins to metabolic control could provide new insights into common human obesity, particularly for individuals with genetic susceptibility to weight gain.

Recent advances including the identification of the BBSome’s role in the regulation of mitochondrial dynamics and function through modulation of the activity of DRP1 are highly significant. Rescuing many BBS phenotypes by correcting mitochondrial defects associated with this condition open new opportunities for the development of novel therapeutics to manage several BBS phenotypes simultaneously. Drugs aimed at improving mitochondrial function or exploiting mitochondrial dysfunction to treat diseases are under development [119, 120]. However, more work is needed to better understand the link between the BBSome and mitochondria function.

## Data Availability

No datasets were generated or analysed during the current study.

## Abbreviations

5-HT2C: Serotonin 5-hydroxytryptamine 2C receptor
AgRP: Agouti-related peptide
BBS: Bardet-Biedl syndrome
BMP8B: Bone morphogenic protein 8B
CNS: Central nervous system
DRP1: Dynamin-like protein 1
EVs: Extracellular vesicles
IFT: Intraflagellar transport
IMCD3: Immortalized collecting duct cell line
GPCR: G protein-coupled receptors
LepRb: Leptin receptor
NPY2R: Neuropeptide Y2 receptor
MCHR1: Melanin concentrating hormone receptor 1
POMC: Proopiomelanocortin
SF1: Steroidogenic factor 1
Stat3: Signal transducer and activator of transcription 3
SSTR3: Somatostatin receptor 3

## Code availability

NA.
